# Tip60-mediated acetylation activates transcription independent apoptotic activity of Abl

**DOI:** 10.1186/1476-4598-10-88

**Published:** 2011-07-22

**Authors:** Zhihua Jiang, Ravindra Kamath, Shunquian Jin, Manimalha Balasubramani, Tej K Pandita, Baskaran Rajasekaran

**Affiliations:** 1 Department of Microbiology and Molecular Genetics (Z.J., R.V., R.B), University Drive Pittsburgh, PA 15240, Pittsburgh PA 15261, USA; 2 Veterans Affairs Health Care, University Drive Pittsburgh, PA 15240, Pittsburgh PA 15261, USA; 3 Pittsburgh Cancer Institute, Pittsburgh, PA 15261, USA; 4 Department of Radiation Oncology, UT SouthWestern Medical Center, Dallas, TX 75390, USA; 5 Genomics and Proteomics Core Laboratories, 3343 Forbes Avenue, Pittsburgh, PA 15260, USA

**Keywords:** DNA damage response, histone acetyl-transferase, c-Abl tyrosine kinase, Apoptosis

## Abstract

**Background:**

The proto-oncogene, c-Abl encodes a ubiquitously expressed tyrosine kinase that critically governs the cell death response induced by genotoxic agents such as ionizing radiation and cisplatin. The catalytic function of Abl, which is essential for executing DNA damage response (DDR), is normally tightly regulated but upregulated several folds upon IR exposure due to ATM-mediated phosphorylation on S465. However, the mechanism/s leading to activation of Abl's apoptotic activity is currently unknown.

**Results:**

We investigated the role of acetyl modification in regulating apoptotic activity of Abl and the results showed that DNA strand break-inducing agents, ionizing radiation and bleomycin induced Abl acetylation. Using mass spectrophotometry and site-specific acetyl antibody, we identified Abl K921, located in the DNA binding domain, and conforming to one of the lysine residue in the consensus acetylation motif (**K**XXK--X3-5--SGS) is acetylated following DNA damage. We further observed that the S465 phosphorylated Abl is acetyl modified during DNA damage. Signifying the modification, cells expressing the non acetylatable K921R mutant displayed attenuated apoptosis compared to wild-type in response to IR or bleomycin treatment. WT-Abl induced apoptosis irrespective of new protein synthesis. Furthermore, upon γ-irradiation K921R-Abl displayed reduced chromatin binding compared to wild type. Finally, loss of Abl K921 acetylation in Tip60-knocked down cells and co-precipitation of Abl with Tip60 in DNA damaged cells identified Tip60 as an Abl acetylase.

**Conclusion:**

Collective data showed that DNA damage-induced K921 Abl acetylation, mediated by Tip60, stimulates transcriptional-independent apoptotic activity and chromatin-associative property thereby defining a new regulatory mechanism governing Abl's DDR function.

## Background

DNA damage response is a highly conserved response elicited by genome damaging agents that ultimately serves to protect genome integrity by inducing cell cycle arrest, DNA repair, or apoptosis [1,2]. Recent studies showed that covalent modification by acetylation, in addition to phosphorylation, regulates the activity of a number of proteins mediating the DDR [3-5]. Principal acetyl-transferases (ATs), implicated in DDR include p300, CREB-binding Protein (CBP), pCAF and the MYST family acetylases,Tip60 (KAT5) and hMOF [5-9]. Consistent with their role in DDR, cells compromised for p300, Tip60 or hMOF function exhibit heightened sensitivity to DNA damaging agents due to defective cell-cycle checkpoint activation, DNA repair and or apoptosis [4,5]. Substrates of DNA damage-responsive acetyl-transferases include both histones and non-histone proteins such as p53, p73α, Ku70, E2-F1 and Sp1 [10-15]. In general, modification of histones by acetylation leads to unfolding of the nucleosomal fiber structure at DNA strand breaks perhaps facilitating access to DNA damage signaling and repair proteins [3,4]. On the other hand, acetylation of non-histone proteins is shown to stimulate their DNA-binding and transactivation function. In the case of p53, acetylation is shown to stimulate DNA-binding, stability and pro-apoptotic activity [10-12]. Similarly, acetylation of Ku70, E2F-1 and p73α selectively stimulates their apoptotic gene-specific transactivation function [13-15]. Interestingly, acetylation of Sp1 is shown to loosen *bak *promoter DNA binding [16]. Acetylation of ATM kinase, mediated by Tip60 complexed with MRE11-RAD50-NBS is shown to stimulate ATM's catalytic function during DNA damage [17]. Loss of acetylation due to depletion of hMOF by RNA interference prevents ATM-mediated phosphorylation of p53 and Chk2 [18]. Altogether, available evidences suggest that acetylation is an important modification that critically governs the activity of DDR proteins.

The ubiquitously expressed Src-related c-Abl tyrosine kinase is a well-documented regulator of cellular responses induced by oxidative stress, DNA damaging agents and growth factors [19-22]. *Abl *null mice display lymhopenia, reduced fertility, osteoporosis, and high neonatal mortality indicating its importance in normal growth and development [23]. Abl contains a large C-terminus whose deletion also caused neonatal lethality in mice signifying the biological role of this domain [24]. In this C-terminus, several discrete functional domains such as DNA binding domain (DBD), nuclear location and export sequences (NLS, NES) and RNA polymerase II-C-terminal binding domain (CTD) have been identified [25-28]. The presence of both NLS and NES is thought to allow nuclear-cytoplasmic shuttling of Abl and gain access to substrates in both compartments during signaling. The DBD of Abl is hyperphosphorylated by cdc2 during mitosis and this correlates with decreased chromatin association [26]. *In vitro *studies showed that Abl binds A/T-rich sequences through HMG-1 like boxes (HLBs) located within its DBD [27]. The RNAP II CTD is required for processive phosphorylation of Pol II largest subunit and for transcription enhancement function of Abl [28]. The catalytic function of Abl is normally tightly regulated through inter/intra-molecular association [20-22] and phosphorylation [29] but stimulated upon exposure to IR or other genotoxic agents (e.g. cisplatin, methyl methane sulfonate, mitomycin C and hydrogen peroxide) [19,20]. Gamma-radiation induced Abl kinase activation is due to S465 phosphorylation mediated by ATM kinase [29]. Kinase-activated Abl, in turn, stimulates the transactivation property of p53 and its family members, p73α and p63α either through direct phosphorylation and/or association [30-33]. Corroborating well with attenuated induction or activation of Abl downstream targets, *Abl^-/- ^*mouse cells display apoptotic-resistant phenotype against a class of genotoxins [21,22]. Phosphorylation of a subset of DNA repair proteins, DNA-PK and RAD9 by Abl is shown to diminish their repair capability [34-36], although phosphorylation of RAD51 enhanced its association with RAD52 and consequently repair [37]. In sum, current data suggests that Abl modulates the cell death response induced by DNA damaging agents through multiple mechanisms involving activation of pro-apoptotic molecules and inactivation of DNA repair proteins.

We investigated whether acetyl modification is stimulatory for Abl's apoptotic function and observed that induction of DSB, either through IR exposure or treatment with belomycin, triggers c-Abl acetylation on K921. The requirement of S465 phosphorylation for K921 Abl acetylation further revealed that ATM-activated Abl is a substrate for DNA damage responsive acetyl-transferase/s. Attenuated apoptosis induced by DSB generating agents in cells expressing K921R mutant irrespective of new protein synthesis demonstrated the modification requirement in stimulating non-transcriptional apoptosis induced by Abl. Reduced chromatin binding displayed by K921R-Abl mutant compared to wild type Abl in IR exposed cells showed that acetylation positively modulates the DBD activity of Abl and may be closely linked to its non-transcriptional apoptotic activity. Finally, the ability of Tip60 to interact with Abl both *in vitro *and *in vivo *identified Tip60 as an upstream Abl modifier in the DNA damage signaling pathway.

## Results

### IR exposure induces acetylation of c-Abl

**Figure 1:**
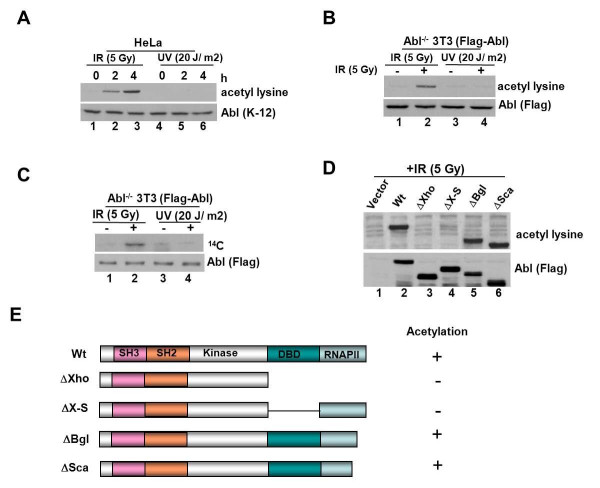
**IR exposure induces c-Abl acetylation**. **(a) **HeLa cells were exposed to 5 Gy of IR or 20 J/m2 of UV and collected at 0, 2 and 4 h later. Anti-Abl immunoprecipitates were prepared using K-12 antibody and resolved on a 5-15% SDSPAGE. The proteins were transferred onto Immobilon-P and immune-analyzed with pan-specific anti-acetyl lysine antibody (*top panel*). The membrane was stripped as described in methods and reprobed with anti-Abl antibody (*bottom panel*). **(b) ***Abl^-/- ^*3T3 cells reconstituted with Flag-tagged Abl were either mock, IR (5 Gy) or UV (20 J/m2) treated and collected 4 h later. Anti-Abl immunoprecipitates were prepared and immune-analyzed with acetyl lysine (*top panel*) or Flag antibody (*bottom panel*). **(c) ***Abl^-/- ^*3T3 cells reconstituted with Flag-tagged Abl were labeled with 14C acetyl CoA for 2 h and then exposed to IR (5 Gy) or UV (20 J/m2) and collected 4 h later. Anti-Abl immunoprecipitates were prepared, resolved on 8% SDS-PAGE, and subjected to fluorography followed by autoradiography (*top panel*). An aliquot of the immune complex was immune-analyzed with Flag antibody (*bottom panel*). **(d) **293 cells were transfected with either vector alone or the indicated Abl constructs shown in *panel ***e**, using lipofectamine. At 36 h post-transfection, cells were exposed to 5 Gy of IR and anti-Flag immunoprecipitates were formed and immune-analyzed with anti-acetyl lysine (*top panel*) or anti-Flag antibody (*bottom panel*). **(e) **Schematic representation of the Abl constructs used in the experimentation described in *panel ***d**.

To determine whether Abl is acetylated post DNA damage, extracts of HeLa cells exposed to either IR (5 Gy) or UV (20 J/m^2^) were formed at 0, 2 and 4 h post-treatment and Abl was immunoprecipitated using anti-Abl (K12) antibody. Western blot analysis of the precipitants with pan-specific anti-acetyl lysine antibody revealed an immunoreactive band of ~130 kDa corresponding to the size of Abl in IR exposed samples (Figure 1a; *lanes *2 and 3, *top panel*). Neither untreated nor UV-treated extracts displayed this immune-reactive band despite comparable presence of Abl protein (*bottom panel*). A similar analysis using Abl^-/- ^3T3 cells reconstituted with Flag-tagged Abl confirmed that the acetyl modification is induced specifically by IR but not UV (Figure 1b). We next radiolabeled Flag-Abl^+ ^3T3 cells with [^14^C] acetyl CoA and exposed to IR. Resolution of Abl immune-complex by SDS-PAGE followed by autoradiography showed a ^14^C-labeled band of ~130 kDa in IR exposed samples that coalesced with Abl band, revealed by immunoblotting (Figure 1c). Samples treated with UV did not display this radiolabeled band. To map the acetylation site, we transfected 293T cells with cDNA constructs of Abl containing truncations or internal deletions (Figure 1e), and then assessed for IR-induced acetyl modification. Wild type, ΔSca and ΔBgl-Abl showed acetylation whereas the DNA binding domain (DBD) deleted mutant constructs ΔXho and ΔXho-Sal did not display this modification (Figure 1d). The data showed that the DBD of Abl harbors the acetyl-modified residue/s.

### K921 of Abl is acetylated in response to DNA damage

To identify the acetyl modified residues, 293 cells transiently expressing Flag-tagged Abl were exposed to IR and Abl was immunopurified, subjected to trypsin digestion, and analyzed by MS. Database searching using sequest identified mouse cAbl (P00520) with high confidence (Additional Files 1 &2). Specific MS/MS analysis of the peptide ion at m/z 477.8 corresponding to the (M+3H)3^+^ species showed a mass of 1431 Da, an increase of 56 Da compared to the calculated mass of peptide without modification. Acetylation of K921 and methylation of Cys899 (44Da + 14Da = 56Da) or carbamidomethylyation of Cys899 could account for the increased mass of the modified peptide; however, if Cys899 (57 Da mass change) is carbamidomethylated, the corresponding Y_7_^+^, Y_8_^+^, and Y_9_^+ ^ions in the spectrum would be assigned to mass peaks that is approximately 1 Da higher in mass. MS/MS data, therefore, points to the possibility of acetylation on K921 with a better match of peaks between observed and calculated mass.

**Figure 2:**
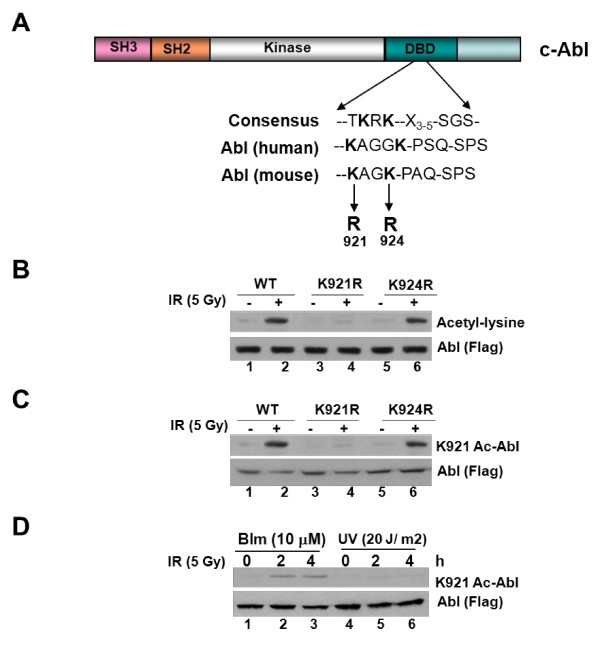
**K921 of Abl is modified by acetylation following DNA strand breaks**. **(a) **Structure of c-Abl with the consensus acetylation sequence (mouse and human) located in the DNA-binding domain (DBD) is shown. **(b) ***Abl*^-/- ^3T3 cells, reconstituted with Flag-tagged WT or K>R (921, 924) mutants of Abl, were either mock-treated or exposed to 5Gy of IR. Anti-Flag immunoprecipitates were prepared and immune-analyzed with acetyl-lysine (*top panel*) or Flag antibody (*bottom panel*). **(c) **Immunoblot, shown in *panel *A, was reprobed against K921 site-specific acetyl-Abl antibody (*top panel*). An aliquot of the lysate was also immune-analyzed with anti-Flag antibody (*bottom panel*). **(d)** Bleomycin induces K921 Abl acetylation. *Abl^-/-^*3T3 cells reconstituted with vector or Flag-tagged Abl were exposed to bleomycin (10 μM; 1 h) and lysates, prepared 4 h later, were subjected to immunoblotting with K921 acetyl Abl (*top panel*) or anti-Flag (*bottom pane*l) antibody.

Located in the DBD (Figure 2a), K921 of Abl represents one of the two lysine residues within a consensus acetylation sequence (**K**AG**K**--PAQ--SPS) found in HAT substrates such as E2F-1, p53 and Ku70 [11-14]. As a logical extension, K921 and K924 of Abl were individually substituted with arginine (R) and tested for acetyl modification by IP followed by immunoblotting against anti-acetyl lysine antibody. K921R mutant was resistant to IR-induced acetyl modification demonstrated by lack of reactivity to acetyl-lysine antibody (Figure 2b). However, reactivity to K924-Abl remained comparable to WT. We next generated K921 site-specific acetyl-Abl antibody by immunizing rabbits with acetylated Abl peptide (described in *Methods*) and evaluated its reactivity to WT and K>R mutant of Abl by reprobing the membrane, shown in *panel *A. Result showed comparable reactivity to WT and K924R mutant of IR-treated cells but least to K921R mutant (Figure 2c). We next immune-analyzed lysates formed from cells treated with DSB-inducing agent, bleomycin and observed acetylation of WT and K924R mutant but not K921R mutant at both 2 and 4 h post-treatment (Figure 2d). Collective data established that DSB signals triggers K921 Abl acetylation.

### S465 phospho-Abl is acetylated post DNA damage

**Figure 3:**
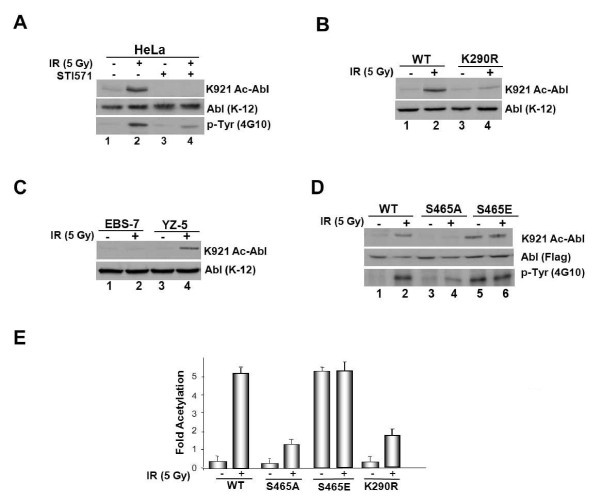
**ATM phosphorylated Abl is targeted for acetyl-modification on K921**. **(a) **HeLa cells were incubated with STI571 (10 μM; 1h) and then either mock treated or exposed to 5 Gy of IR. At 4 h post-treatment, lysates were prepared, adjusted for equal protein content and immune-analyzed with K921 Ac-Abl antibody (*top panel*) or K-12 Abl antibody (*middle panel*). Anti-Abl immunoprecipitates were also prepared and probed with anti-pTyr antibody (*bottom panel*). **(b) ***IR-induced acetylation of WT and Kinase-defective Abl mutant*. Abl-null 3T3s reconstituted with WT or kinase-defective Abl (K290R) were exposed to IR (5 Gy) and lysates were formed and subjected to immunoblot analysis with K921 Ac-Abl antibody. **(c) **Mock- and IR-treated ATM-deficient(EBS-7) and ATM-proficient (YZ-5) fibroblasts were collected at 4 h post-treatment and lysates were formed, adjusted for equal protein concentration, and subjected to immunoblotting with K921 Ac Abl (*top*) or anti-Abl (*bottom*) antibody. **(d) **293T cells were transfected with WT, S465A or S465E mutant of Abl cDNA. At 36 h post-transfection, cells were exposed to IR (5 Gy) and lysates were formed 4 h later and subjected to immunoblotting with K921 Ac Abl (*top panel*) or anti-Flag (*middle panel*) or anti-pTyr (4G10) antibody (*bottom panel*). **(e) **Fold increase in acetylation was assessed by densitometric analysis of the immune-reactive band using phosphor-imager and the data are presented as means ± SD, *n *= 3.

ATM-mediated phosphorylation on residue S465 stimulates Abl kinase activity upon IR exposure [29]. To study the interplay between kinase activation and acetylation, we tested the effect of Abl kinase inhibitor, imatinib (*gleevec*) on IR-induced K921 Abl acetylation. Immune-analysis of the lysates with K921-Ac-Abl antibody revealed that the inhibitor blocked K921 Abl acetylation induced by IR (Figure 3a, *compare lane 4 *to *2*). Reduced p-Tyr content on Abl in inhibitor-treated cells compared to control treatment, revealed by anti-p-Tyr (4G10) immunoblotting confirmed Abl kinase inhibition (*bottom panel, compare lane 4 to 2*). A similar result was obtained with the kinase-inactive Abl mutant (K290R) in that the mutant showed decreased K921 acetylation compared to WT after IR treatment (Figure 3b). Examination of Abl acetylation in ATM-proficient (YZ-5) and deficient (EBS-7) fibroblasts showed that the modification induced by IR is dependent on ATM function (Figure 3c, l*ane *2). To determine whether ATM phosphorylated Abl is subject to acetyl modification we next examined modification on the phosphorylation-resistant S465A and the constitutively active S465E mutant of Abl. Whereas WT and S465E-Abl mutant displayed K921 Abl acetylation, S465A mutant showed very little acetylation (Figure 3c, *lanes *2 and 6). S465E mutant displayed acetylation in the absence of IR that was further enhanced after γ-irradiation by ~5.53 fold (Figure 3d, *lanes *5 and 6). Taken together, the results showed that ATM phosphorylated S465-Abl is acetyl modified on K921 post DNA damage.

### Identification of Tip60 as a predominant Abl-specific acetylase

**Figure 4:**
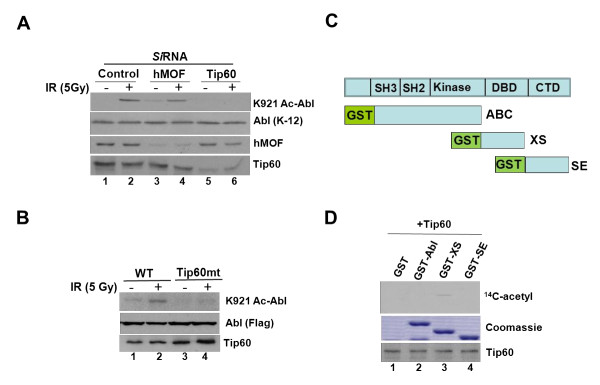
**K921 Abl acetylation is mediated by Tip60 acetyl-transferase**. **(a) **Human colorectal cancer cells (HCT116+Ch3;H3) were transfected with siRNA specific for Luciferase, Tip60 or hMOF and then mock treated or exposed to IR (5 Gy) at 36 h post-transfection. Lysates were formed at 4 post-treatment, normalized for equal protein content, and immune-analyzed with K921 Ac Abl or Abl antibody. An aliquot of the lysate was also subjected to immunoblotting with anti-Tip60 or hMOF antibody. **(b) **293T cells were transfected with plasmids carrying WT or HAT-defective Tip60mt cDNA, and at 36 h post-transfection, cells were mock-treated or exposed to 5 Gy of IR. Lysates were formed 4 h later and subjected to immunoblotting with K921 Ac Abl (*top panel*), anti-Flag (*middle panel*) and anti-Tip60 antibodies, respectively (*bottom panel*). **(c) **Structural domains of GST-Abl fusion products (Abl-ABC, Abl-XS and Abl-SE). **(d)***Tip60 acetylates c-Abl in vitro*. GST-fusion proteins, shown in *panel *C, were expressed in bacteria purified, and incubated with Tip60 immunoprecipitates prepared from IR (5 Gy)-treated HeLa cells in the presence of ^14^C-acetyl CoA in HAT buffer. The reaction products were resolved in 5-15% SDS-PAGE and the gel was dried and exposed to autoradiography (*top panel*). Coomassie blue staining of the GST-fusion products (*middle panel*). Anti-Tip60 immunoblotting (*bottom panel*).

In an attempt to identify the acetylase responsible for modifying Abl, we initially examined the requirement of p300 HAT. However, comparable K921-Abl acetylation in HCT116/p300^- ^cells containing targeted deletion of exon 2 of *EP300 *gene [7] and in the parental HCT116 cell line demonstrated p300 dispensability (Additional File 3). We next tested the contribution of MYST-family acetyl-transferases, Tip60 and hMOF by transfecting HeLa cells with synthetic siRNA specific for either hMOF or Tip60. After confirming reduction of their expression by immunoblotting (Figure 4a), the transfectants were exposed to 5 Gy of IR and Abl acetylation was assessed. Result showed that the modification was partially inhibited in hMOF-suppressed cells but was completely abrogated in Tip60-suppressed counter parts (Figure 4a, *top panel*, *compare lane *4 and 6 to 2). To further evaluate the involvement of Tip60, we co-expressed Abl with either WT or HAT-defective mutant of Tip60 (Q377E/G380E) in 293T cells and immune-analyzed the lysates for K921 Abl acetylation. WT Tip60 expressing population displayed modest acetylation that was greatly enhanced after γ-irradiation (Figure 4b, *top panel*, compare lane 1 to 2). In contrast, cells expressing Tip60 mutant displayed diminished IR-induced Abl acetylation (lane 4). Anti-Tip60 (HA) immunoblotting confirmed comparable WT and Tip60mtexpression in these transfectants (Figure 4b, *bottom panel*). To determine whether Abl is directly acetylated by Tip60 we performed *in vitro *HAT assays using GST fragments carrying various domains of Abl as substrates (Figure 4c). Incubation of Abl fragments with Tip60 immunopurified from γ-irradiation-treated HeLa cells in the presence of ^14^C-acetyl CoA resulted in labeling of the GST Xho-Sal fragment containing the DBD of Abl (Figure 4d, *lane *3, *top panel*), in agreement with the presence of K921 residue in this domain. Neither GST-ABC nor GST-SE displayed ^14^C labeling. Coomassie blue staining showed comparable substrate in each of these HAT reactions (*middle panel*). Similarly, anti-Tip60 immunoblotting confirmed the presence of equivalent levels of Tip60 in each of the HAT reactions (*bottom panel*). Together, the findings demonstrated that Tip60 acetylates Abl in response to DNA damage although a role for hMOF cannot be completely ruled out.

### c-Abl co-precipitates with Tip60 acetylase following DSB induction

**Figure 5:**
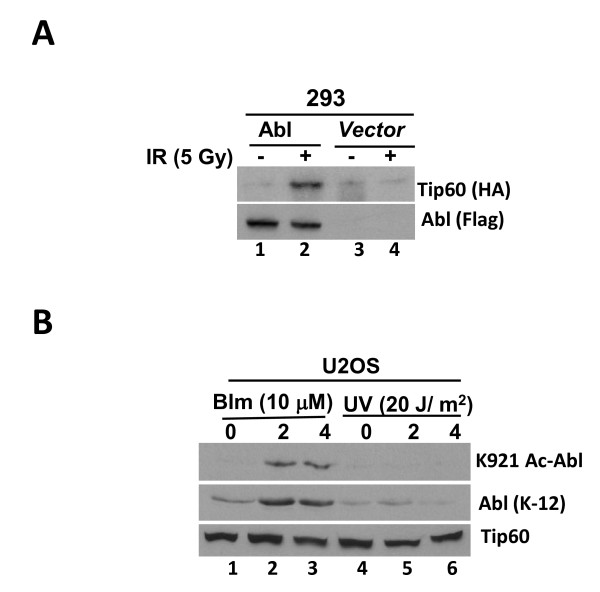
**Co-precipitation of Abl with Tip60 following DSB induction**. **(a) **293 cells were transfected with pcDNA3.2 plasmids carrying Flag-Abl and HA-Tip60 cDNA using lipofectamine. At 36 h post-transfection, cells were exposed to 5 Gy of IR 4 h later lysates were formed, adjusted for equal protein content and subjected to immunoprecipitation with anti-HA antibody. Immunoprecipitates were resolved on SDS-PAGE and subjected to western blotting against anti-Flag (*top panel*) or anti-HA antibody (*bottom panel*). **(b) **Abl-null 3T3 cells reconstituted with vector alone or Flag-tagged WT-Abl were exposed to bleomycin (10 μM) and collected 0, 2 and 4 h later. Lysates were formed, adjusted for equal protein concentration and subjected to immunoprecipitation with anti-Flag (Abl) antibody. The immune-complexes were resolved on SDS-PAGE, transferred onto Immobilon-P and probed with anti-HA (Tip60) or anti-Flag (Abl) antibody.

To examine whether c-Abl associates with Tip60 *in vivo *we performed co-immunoprecipitation assays using lysates prepared from Flag-Abl and HA-Tip60 transfected 293 cells. Result showed the presence of Abl in anti-Tip60 (HA) immunoprecipitates prepared from extracts of IR-but not mock-treated cells (Figure 5a). No association was observed in UV-treated cell lysates. When endogenous interactions between Tip60 and Abl was assessed in reconstituted *Abl^+^*3T3 cells we observed that cells treated with bleomycin (Figure 5b) showed enhanced association than observed with control treatment. The findings allow us to conclude that DNA damage enhances association between Abl and Tip60 consequently facilitating acetylation.

### K921 acetylation is required for Transcription-independent apoptosis mediated by Abl

**Figure 6:**
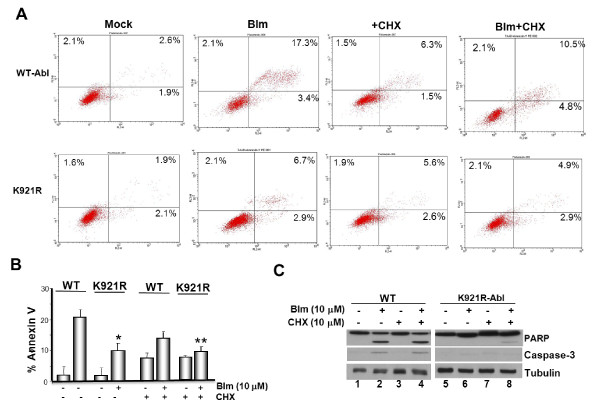
**Acetylation of Abl on K921 is required for transcription-independent apoptosis triggered by DNA strand breaks**. **(a) ***Abl^+ ^*3T3 cells expressing WT or K921R Abl mutant were pre-treated with 100 μg/ml of CHX or vehicle for 1 h and then exposed to 10 μΜ bleomycin (Blm) for 1 h. Cells were collected 48 h later, stained with Annexin V/PI and analyzed by flow cytometry. **(b) **Percent AnnexinV/PI-positive and cell population shown in *panel *A is graphed. Mean value obtained from three independent experiments with SD is given. *P < 0.01; **P < 0.05. **(c) **Lysates of mock and Blm-treated cells (10 μM; 1 h), shown in *panel *A, were adjusted for equal protein content and immunoblotted against PARP (*top panel*), caspase-3 (*middle panel*) or tubulin antibody (*bottom panel*).

Given that acetylation activates the pro-apoptotic activity of E2-F1, p53 and p73α [12-14] we examined whether acetylation stimulates Abl's apoptotic function. For this purpose, cells reconstituted with WT or the non-acetylatable K921R-Abl mutant were exposed to 10 μΜ of belomycin and cell death was assessed at 48 h post-treatment. Annexin V/PI staining followed by flow cytometric analysis revealed ~20.7% apoptosis (early and late) in cells expressing WT Abl (Figure 6a). Under similar conditions, % apoptosis in cells expressing K921R mutant was ~9.6, a number that represents ~2 fold reduction compared to WT. When the results of three independent experiments were combined, cells expressing the K921R mutant showed a statistically significant (*P < 0.01) reduced apoptosis compared to WT (Figure 6b) consistent with acetylation as a required modification for activating Abl's cell death-inducing ability. To determine whether K921 acetylation promotes transcription-dependent or independent apoptosis, we included the *de novo *protein translation inhibitor, cyclohexamide in the assay. Although the inhibitor was slightly toxic demonstrated by high basal levels of cell death inWT and K921R mutant (~7.8% and ~8.2%, respectively) it neither affected Abl expression nor K921 acetylation (Additional File 4). Nonetheless, when bleomycin-induced cell death was assessed in the presence of CHX, % cell death in WT-Abl cells was slightly reduced (~15.3%) compared to that observed in the absence (~20.7%). K921R mutant showed significantly reduced cell death both in the absence or absence of the inhibitor (~7.8% vs ~9.6%). Attenuated apoptosis displayed by the K921R mutant irrespective of new protein synthesis demonstrated a requirement for Abl acetylation in triggering transcription-independent cell death. Correlating well these results, immunoblot analysis of the lysates prepared from these panel of cells with anti-PARP and anti-caspase-3 antibody (that recognizes cleaved fragment) showed comparable cleavage in bleomcyin-treated WT Abl cells in the presence or absence of CHX (Figure 6c, *compare lane 2 and 4*). Lysates of K921R mutant cells showed reduced but comparable cleavage irrespective of inhibitor treatment, consistent with attenuated cell death (*compare lane 6 to 7*).

**Figure 7:**
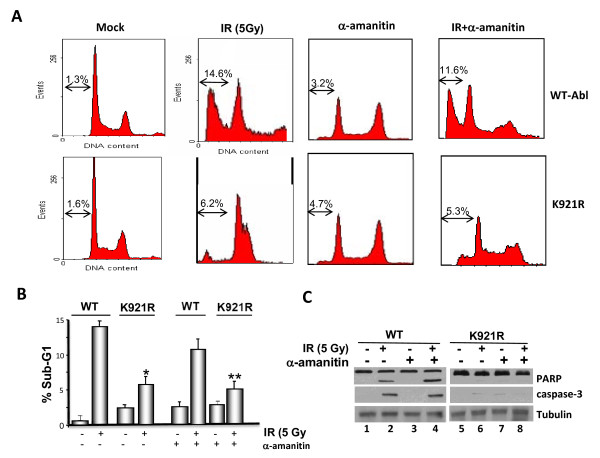
**Restoration of WT but not K921R mutant of Abl reinstates IR-induced transcriptional-independent apoptosis**. **(a) **HeLa cells stable depleted for Abl expression were transfected with either WT or K921R-Abl mutant along with GFP cDNA constructs. 36 h post-transfection, cells were incubated with 100 μg/ml of α-amanitin or vehicle for 2 h and then either mock-treated or exposed to 4.3 Gy of IR. 48 h later, cells were then stained with PI and subjected to flow cytometry. **(b) **Percent sub-G1 DNA containing GFP+ cell population is graphed and mean value obtained from three independent experiments with SD is given. *P < 0.01; **P < 0.01. **(c) **Lysates of mock and BLM-treated cells were adjusted for equal protein content and subjected toimmunoblotting with anti-PARP (*top panel*) or anti-caspase-3 antibody (*middle panel*). Anti-tubulin immunoblotting shows equal protein loading (*bottom panel*).

To reinforce the results of the above analysis, HeLa cells stable depleted for Abl expression by shRNA (described in our previous publication Ref: 44) were transfected with WT or K921R mutant of Abl, along with transfection marker, GFP. At 36 h post-transfection, cells were exposed to 4.3 Gy of IR, a dose that triggers optimal cell death in these cell lines, and cell death was assessed by flow cytometric analysis of cells gated for GFP+ population (Figure 7a and 7b). Determination of % sub-G1 DNA containing population revealed reduced cell death in K>R mutant (~6.2%) compared to WT-Abl expressing cells (~14.6%). When cell death was assessed in the presence transcription inhibitor, α-amanitin, a RNA polymerase II poison, we observed slightly reduced cell death in WT-Abl cells (~11.6%) compared to control treatment (~14.6%). However, K921R mutant showed similar levels of cell death both in the presence and absence of the inhibitor (~6.2% vs ~5.3%). Consistent with these results, comparably diminished PARP and caspase-3 cleavage was observed in lysates formed from K921R expressing cells with or without α-amanitin (Figure 7c). Taken together, the findings reinforced the involvement of Abl, and more importantly, K921 Abl acetylation requirement in promoting transcription-independent apoptosis triggered by DSB-inducing agents.

### K921 acetyl-Abl is enriched in chromatin fraction of IR-treated cells

**Figure 8:**
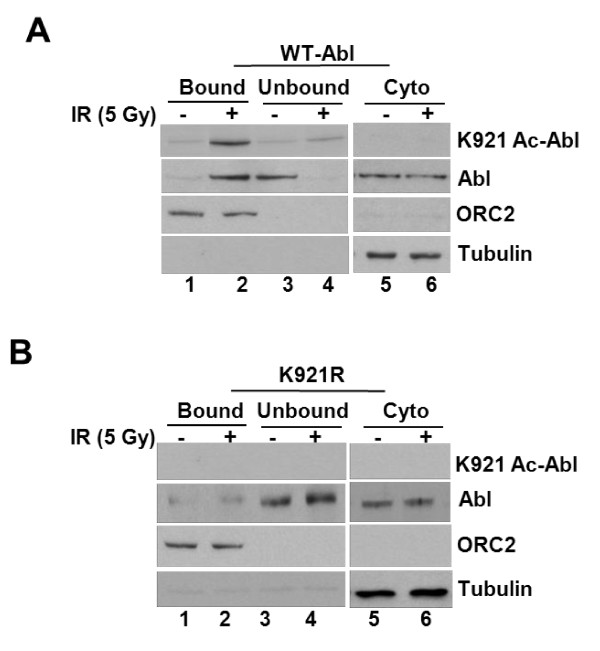
**Enrichment of K921 Acetylated Abl in chromatin fractions of γ-irradiated cells**. **(a) **Reconstituted *Abl^+ ^*3T3 cells were exposed to IR (5 Gy) and at 18 h post-treatment, cells were harvested, lysed in hypotonic buffer and fractionated into nuclear and cytoplasmic fraction as described by Méndez *et al *[46]. Nuclear fraction was again separated into chromatin bound and unbound and subjected to immunoprecipitation with anti-Abl (K-12) antibody. The immune-complex was analyzed by subjecting to western blotting with K921 Ac-Abl or Abl antibody (*top panels*). Input lysates were also blotted with tubulin (cytoplasmic marker) or ORC2 (chromatin marker) antibody (bottom panel). **(b) ***Abl^-/- ^*3T3 cells reconstituted with K921R mutant of Abl were exposed to 5 Gy of IR and nuclear and cytoplasmic fractions were formed and immune-analyzed for K921 Ac-Abl, Abl, tubulin or ORC2 proteins as described in *panel ***a**.

Abl association with chromatin is negatively regulated during mitosis by cdc2-mediated phosphorylation on multiple ser/thr residues, located in the DBD of Abl [26]. To determine whether Abl acetylation on K921 affects its chromatin-associative property, we compared the presence of acetyl-Abl in chromatin fractions of mock and IR exposed cells. Extracts formed from mock- and IR-treated *Abl^+ ^*3T3 cells were separated into nuclear and cytoplasmic fractions as described in *Methods *and their integrity was confirmed by immunoblotting with chromatin marker, Orc2 and cytoplasmic marker, tubulin (Figure 8a). Nuclear fractions were then divided into equal aliquots and immunoprecipitated with either K921 Ac-Abl or Abl antibody. Anti-Abl immunoblotting showed increased presence of both acetyl-Abl and total Abl in chromatin fractions of IR-treated extracts compared to mock-treated samples (*top panel, compare lane 2 to 1*). Cytoplasmic fractions showed no alterations in Abl content between mock- and IR-treated cells (bottom panels, *lanes 5 *and *6*). K921R-Abl mutant showed a lack of enrichment in the chromatin fraction following irradiation (Figure 8b). Levels of K921R Abl in the unbound fractions of mock- and IR-treated cells remained unchanged. The impaired chromatin association displayed by the K921R mutant compared to WT in IR-treated cells suggests that acetyl modification positively modulates Abl's chromatin-binding activity and may be closely linked to its non-transcriptional apoptotic activity.

## Discussion

Induction of apoptosis is a central function of c-Abl in the DNA damage signaling pathway; however, the mechanism/s leading to activation of its apoptotic activity is not known. While previous studies established that the kinase function of Abl is essential for its cell death-inducing function [20-22] and is activated by phosphorylation, the present study demonstrates that acetylation activates Abl's apoptotic activity. Specifically, our study shows that K921 of Abl, located within the DNA binding domain, and confining to one of the lysine in the consensus acetylation motif identified in pro-apoptotic molecules such p53, E2F-1 and p73α [12-15] is modified by acetylation during DNA damage. Acetylation selectively activates the non-transcriptional cell death induced by Abl as the non-acetylatable K921R mutant displayed attenuated apoptosis compared to WT in the absence of new protein synthesis. Decreased chromatin binding displayed by the acetyl-resistant K>R mutant compared to wild-type supports the argument that acetyl modification positively modulates chromatin-binding property of Abl and may be closely linked to its apoptotic activity. Finally, our observation that Tip60 associates with and acetylates Abl both *in vitro *and *in vivo *identified Tip60 as a predominant DNA damage responsive acetylase that modifies Abl on K921.

Results of the present study raise an interesting question about how DNA damage induces K921 acetylation and activates its apoptotic activity. Acetylation of pro-apoptotic molecules such as p53 and p73α activates selective transactivation function towards apoptotic genes, *bax *and *puma *through enhanced promoter DNA binding [11,15]. On the other hand, acetylation of Sp1 is shown to loosen binding to *bak *promoter consequently allowing access of Sp3 to the promoter region to drive *bak *transcription [16]. However, unlike p53, p73α and Sp1, c-Abl is not known to bind specific DNA sequence. Instead, *in vitro *DNA binding studies showed that Abl binds A/T rich DNA sequences through its HMG-1 like boxes (HLBs), located within its DBD [27]. David and coworkers [38] further reported that Abl binds DNA sequences containing AAC motifs and to distorted DNA structures. HMG proteins such as HMG1A and HMGI/Y are shown to bind A/T-rich stretches of chromatin through AT-hooks and contribute to chromatin remodeling [39-41]. Moreover, HMG proteins have been reported to displace DNA repair molecules from chromatin allowing the access of DNA caspases and nucleases [40-42]. Phosphorylation of a subset of DNA repair proteins, Rad9 and DNAPK by Abl is documented to suppress their repair capability [34,36]. When taken together, the findings lead us to speculate that acetylated Abl induces transcription-independent apoptosis through binding to AT/AAC rich sequences in chromatin facilitating remodeling and or inhibiting DNA repair. Another intriguing possibility is that Abl may promote apoptosis through increased Bad/Bax protein accumulation. In the cytoplasm, pro-apoptotic proteins such as Bax and Bad are kept inactive through interactions with 14-3-3 proteins. Yoshida *et al *[43] showed that JNK-mediated phosphorylation of 14-3-3 dissociates 14-3-3/c-Abl complex consequently leading to increased nuclear accumulation of c-Abl. Conceivably, nuclear retention of acetyl modified Abl through chromatin association may lead to reduced availability of Abl in the cytoplasm consequently allowing accumulation of Bad and Bax in the cytoplasm and enhancing cell death. Additional studies, however, are required to fully uncover the precise mechanism by which Abl promotes transcriptional-independent apoptosis.

The mechanism by which DNA damage induces Abl acetylation by Tip60, at present, remains speculative. The lack of acetyl modification on DNA binding domain deleted mutants of Abl (ΔXho-Sal and ΔXho) indicates a requirement and an involvement of this domain for the modification. In addition, Abl requires ATM phosphorylation and residue (S465) for the modification since the phosphorylation resistant mutant, S465A-Abl failed to undergo acetylation upon exposure to IR and bleomycin. Moreover, the kinase-defective K290R-Abl was resistant to acetylation triggered by these DSB-inducing agents. We speculate that Abl either binds DNA lesion directly through its DBD or recruited to the lesion as part of ATM complex for interaction and acetylation by Tip60.Acetylation does not influence Abl kinase activity since activation was observed within 1 h post IR and acetylation was observed 2 h later. Moreover, mutants of Abl that cannot be activated (S465A-Abl and K290R-Abl) are resistant to acetylation induced by IR. The constitutively active S465E-Abl mutant, on the other hand, displayed K921 acetylation that was further enhanced after irradiation. These observations suggest that acetylation is a secondary modification that occurs on kinase-activated Abl.

Although Tip60 is essential for K921 Abl acetylation, the current study does not preclude hMOF acetylase in mediating the modification since hMOF-suppressed cells showed partially abrogated acetylation. Cross-talk between Tip60 and human MOF has been reported [9]. Moreover, hMOF has been reported to influence ATM activation in response to DNA damage as depletion of hMOF prevents ATM-mediated phosphorylation of p53 and Chk2 [18]. Such findings impart a role of hMOF in acetylating Abl in response to DNA damage, in addition to Tip60. It is interesting to note that pro-apoptotic proteins such as p53 and E2F-1 are initially modified by phosphorylation, and at latter time-point, by acetylation. Whereas phosphorylation of p53 leads to stabilization, enhancement of promoter DNA binding and activation of its cell-cycle arrest function, acetylation is shown to positively modulate its apoptotic activity. Clearly, a parallel can be drawn between Abl and these DDR proteins in terms of regulation of their activity by coupled process of phosphorylation/acetylation. Specifically, when taken with the previous findings that observation that Abl contributes to growth arrest response [45], and is activated by ATM-mediated phosphorylation upon irradiation [29] lead us to speculate that combined modification by phosphorylation and acetylation modulates the cell growth and apoptotic activity of Abl following DNA damage.

Significance of the current study can be realized from the critical role that Abl plays in mediating the apoptotic response to chemotherapeutic agents that inflict genome damage [20-22]. A recent study showed that activated endogenous Abl kinase dramatically promotes breast cancer cell proliferation and anchorage-independent growth in serum, as well as survival following nutrient deprivation [46]. Additionally, as a co-activator of Androgen receptor (AR), Tip60 contributes to the progression of chemotherapeutic-resistant prostate cancer cell proliferation [47]. Given these observations, the identification of Abl as a Tip60 substrate may be a useful therapeutic tool for developing novel therapeutics in the treatment of cancer.

## Conclusion

In summary, our results reveal a new regulatory mechanism controlling the apoptotic activity of Abl kinase during DNA damage. Specifically, our data shows that K921 Abl acetylation, mediated by Tip60, is a relevant post-translational modification that stimulates the transcription-independent apoptosis induced by DSB. The positive influence exerted by acetylation on chromatin binding function of Abl suggests a link to promoting its apoptotic activity independent of transcription.

## Methods

### Cells

293T and HeLa cells were obtained from ATCC and cultured in DMEM containing 15% heat-inactivated fetal bovine serum and 5% penicillin/streptomycin. Cells were grown in a 5% CO2 incubator at 37°C. EBS-7 and YZ-5 cells were cultured as described [29]. HCT116+ch3 (H3) cells were cultured as described [44]. HCT116/p300^- ^and the parental HCT116 cell line, a generous gift from Dr. Caldas (Johns Hopkins, Baltimore), were grown and maintained as described [7].

### Antibodies

Anti-Tip60 (07-038) and anti-p-Tyr (4G10) antibodies were obtained from Millipore, MA. Pan-specific acetyl-lysine (C4; sc-8663), monoclonal anti-p53 (D01; sc-126), anti-p300 (sc-585) and tubulin antibodies were obtained from Santa Cruz Biotech Company. HRP-conjugated secondary antibodies were purchased from Novus Biologicals (Littleton, CO).K921 site-specific acetyl Abl antibody was generated through commercial contract with Open Biosystems, Inc (Thermo Scientific). In brief, rabbits (2) were immunized with the acetylated c-Abl peptide (LKPAPPPPPACTG**K**acAG) and anti-sera from the immunized rabbits were subjected to affinity purification using the acetylated peptide column.

### SiRNA-mediated ablation of Tip60 and hMof

Synthetic siRNAs specific for Tip60 5'AAAUCUCAUUGCCUGGAGGAUGUCG3' (sense);5AUAGUACAGUGUCUUAUGGUCAAGG-3' (sense) and (hMOF-1,GUGAUCCAGUCUCGAGUGA; hMOF-2, AAAGACCAUAAGAUUUAUU; hMOF-3 CAAGAUCACUCGCAACCAA; hMSL3-1, CGGUUAGUGAAACUUCCAU; hMSL3-2, AAAGGUGACUUCGUCUAAA; control, CACGTACGCGGAATACTTCG, sense strand)were purchased from MWG Biotech. The human colorectal cancer cell line, HCT116+Ch3 (**H3**) was transfected with siRNA using Lipofectamine 2000 (Invitrogen) according to manufacturer's protocol and inhibition of protein expression was confirmed by immunoblotting.

### Construction of c-Abl mutants and Site-directed mutagenesis

Construction of murine c-Abl (type IV) internal and truncated mutants was described in a previous study [28]. Site-specific Abl mutations (K921R, K924R) were created using PCR-based Quickchange kit from Stratagene. The oligonucleotides and its complementary reverse oligo used as primers for K921R is 5'*g cctcctcctgcctgcacagggcgcgcaggcaagcccgca cag agc*3'/5' *gctctgtgcgggcttgcctgcgcg ccc tgtgcaggcaggaggagg c 3'*. For K924R mutagenesis: 5' *cctgcctgcacaggaaaagcagggcgccccgca cag agc ccc agccaa g *3'/5' *c ttggctggggctctgtgcggggcg ccc tgcttttcctgtgcaggcagg*3'. PCR was carried out using primers (0.1 μmol/L) of primers, 2.5 units Taq DNA polymerase, and 3 to 15 ng plasmid DNA. The 1.9-kb BssH1-Kas1 fragments containing the desired mutations were subcloned into pCDNA 3.1 minus vector containing full-length WT Abl cDNA. All constructs were verified by direct DNA sequencing.

### Immunoprecipitation and Immunoblotting

Cells were exposed to either IR (5 Gy), bleomycin (10 μM) or UV (20 J/m2) and harvested at the indicated time points. Cells were washed twice with ice-cold PBS and lysed in 1× lysis buffer containing 10 mM Tris-HCl, pH 8, 240 μΜ NaCl, 5 μΜ EDTA, 1 μΜ dithiothreitol (DTT), 0.1 μg/ml of PMSF, 1% Triton X-100, 1 μΜ sodium vanadate and 1 μM leupeptin, pepstatin, aprotinin by incubation at 4°C for 20 min. Lysates were cleared by centrifugation and the supernatants were normalized for equal protein concentration using BioRad protein assay as per manufacturer's instructions. Lysates were resolved on a 5-12% gradient SDS-PAGE, transferred onto Immobilon-P (Millipore) membrane and probed with the indicated antibody. Equal protein loading was confirmed by anti-tubulin immunoblotting. For reprobing, the membrane was incubated in stripping buffer containing 65 μΜ of β mercaptoethanol, 2% SDS, 50 μΜTris-HCl [pH 6.7] at 55°C for 30 min and then used for immunoblotting after blocking.

### Mass spectrometry

293 cells transfected with Flag-tagged Abl (mouse Type1V) cells were exposed to 5 Gy of IR and harvested 4 h later. Cells were lysed in 1× lysis buffer (described above) and incubated with the anti-Flag monoclonal antibody-conjugated M2 agarose beads (Sigma). The bound material was resolved on a 5-15% SDS-PAGE and the Abl band corresponding to ~130 kDa was excised, incubated with trypsin, and the products were run through a Thermo Finnigan LCQ DECA XP Plus ion trap mass spectrometer interfaced with a Shimadzu Binary HPLC to carry out liquid chromatography-tandem mass spectrometry (LC-MS/MS) analysis.

### HAT Assays

Anti-Tip60 immune-complexes were obtained by incubating lysates formed from mock and irradiated HeLa cells with 5 ul of the antibody as described above. The immune-complex was washed twice in HAT assay buffer (50 μΜ Tris, pH 8.0/10% glycerol/0.1 μΜ EDTA/1 μΜ DTT) and incubated with GST-Abl fusion proteins (0.5 μg) in the presence of 0.5 μCi [acetyl-1-14C] (370 kBq) and cold Acetyl CoA (10 μM). The reaction was carried out in 60 μl of HAT buffer for 30 min at 30°C and terminated using 3× SDS sample buffer. Reaction mixture was resolved on a 5-15% SDS-PAGE, transferred onto Immobilon-P and exposed to autoradiography after flurography.

### Chromatin Fractionation

Chromatin fractionation was performed as described by Mendez and Stillman [48] with minor modifications. Approximately 1 × 106 cells were transfected with either Flag-tagged WT or K>R mutant of Abl using 3T3 Transfection reagent from Mirus, WI. At 36 h post-IR, cells were exposed to 5 Gy of IR and collected 4 h post-treatment. Cells were washed with PBS and resuspended in 200 μL of Buffer A containing 10 μΜ Hepes, 10 μΜ KCl, 1.5 μΜ MgCl2, 0.34 M sucrose, 10% glycerol, 1 μΜ NaF, 1 μΜ Na2VO3, 0.1%Triton X-100, and protease inhibitors. Cells were incubated on ice for 5 minutes, and cytoplasmic proteins were separated from nuclei by low-speed centrifugation at 1,200 ×*g *for 5 minutes. Nuclei were washed with Buffer A and lysed in 200 μL of Buffer B containing 3 μΜ EDTA, 0.2 μΜ EGTA, 1 μΜ DTT, and protease inhibitors followed by incubation on ice for 10 minutes. The lysate was centrifuged at 1,700 × *g *for 4 min. to separate soluble nuclear proteins from chromatin. The isolated chromatin was washed with Buffer B and pelleted by centrifugation at 10,000 ×*g *for 1 minute. Chromatin was resuspended in SDS-sample buffer and sheared by sonication and resolved on 5-15%SDS-PAGE. The proteins were transferred onto Immobilon-P and western blotted against the indicated antibody.

### Apoptosis Assays

For quantitation of apoptosis, cells were stained with Annexin V-FITC2 (Phamingen) and analyzed by FACS Calibur flow cytometer. Approximately, 10,000 total events were collected and then subsequently analyzed for the percentage of Annexin v-FITC (FLH1-H)-positive, propidium iodide (FL2-H)-negative cells.

### Statistical Analysis

Data are expressed as means ± SD and obtained by combining data from separate experiments. Differences between control and treated groups were analyzed using *t *test (two-tailed, unpaired). Differences with a *P *value of <0.05 were considered statistically significant.

## Abbreviations

ATM: Ataxia Telangiectasia Mutated; IR: ionizing radiation; UV: ultraviolet radiation; shRNA: short hairpin RNA; HLB: HMG-1 like box.

## Competing interests

The authors declare that they have no competing interests.

## Authors' contributions

ZJ and RK performed most of the experiments and helped in the preparation of the manuscript. SJ helped in site-directed mutagenesis and flow cytometry. TKP helped through reagents, discussions and editing of the manuscript. RB conceived the study, participated in its design and coordination, prepared the manuscript and supervised the project. All authors read and approved the final manuscript.

## Supplementary Material

Additional file 1 **MS analysis of trypsin digested products of Abl**. **A**. Flag-Abl immunopurified from lysates of IR (5Gy) exposed cells were subjected to in gel digestion following resolution by SDS-PAGE. The trypsin digested products were subjected to MS analysis on an LCQ Deca XP Plus. Click here for file

Additional file 2 **MS/MS of the peptide ion at m/z 477.8, corresponding to the (M+3H)3+ species is shown**. Click here for file

Additional file 3 **p300 acetyl-transferase is dispensable for IR-induced K921 Abl acetylation**. Mock-treated and IR (5 Gy) exposed HCT116 and HCT116/p300^-/-^cells were harvested 4 h later. Lysates were adjusted for equal protein content and probed with K921 site specific anti-acetyl-Abl (*top panel*), anti-Abl (*middle panel*) or anti-p300 antibody (*bottom panel*). Click here for file

Additional file 4 **Effect of CHX and **α**-amaninitin on IR-induced Abl acetylation**. *Abl^+ ^*3T3 cells were exposed to IR (4.3Gy) in the presence or absence of CHX or α-amanitin and harvested at the indicated time points. Lysates were probed with K921 site specific acetyl-Abl (*top panel*) or Abl (Flag) antibody (*bottom panel*). Click here for file
