# Supplementary material for: Tip60-mediated acetylation activates transcription independent apoptotic activity of Abl
Source: Mol Cancer. 2011 Jul 22;10:88. doi: 10.1186/1476-4598-10-88 (PMC3157453; doi:10.1186/1476-4598-10-88)
Supplement: Additional file 1 — MS analysis of trypsin digested products of Abl. A. Flag-Abl immunopurified from lysates of IR (5Gy) exposed cells were subjected to in gel digestion following resolution by SDS-PAGE. The trypsin digested products were subjected to MS analysis on an LCQ Deca XP Plus. [file 1476-4598-10-88-S1.PPTX]

## Slide 1
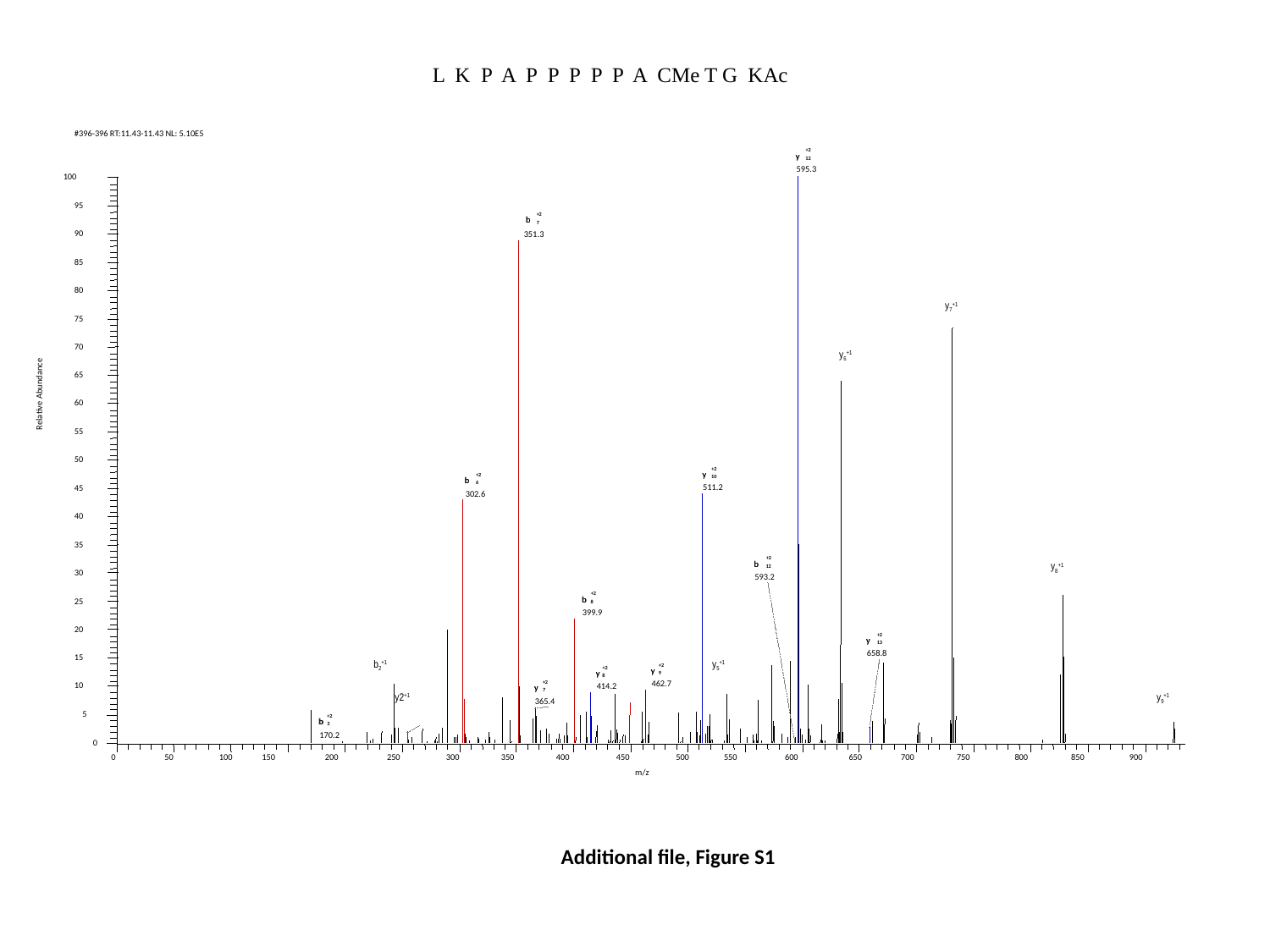

L K P A P P P P P A CMe T G KAc
#396-396 RT:11.43-11.43 NL: 5.10E5
0
0
50
100
150
200
250
300
350
400
450
500
550
600
650
700
750
800
850
900
m/z
+2
y
y
12
595.3
100
95
+2
b
b
7
90
85
80
75
70
65
Relative Abundance
60
55
50
45
40
35
30
25
20
15
10
5
351.3
y7+1
y6+1
+2
y
y
+2
10
b
b
6
511.2
302.6
y8+1
+2
b
b
12
593.2
+2
b
b
8
399.9
+2
y
y
13
658.8
b2+1
y5+1
+2
+2
y
y
y
y
9
8
462.7
+2
414.2
y
y
y2+1
y9+1
7
365.4
+2
b
b
3
170.2
Additional file, Figure S1
