# Supplementary figures and images for: Tip60-mediated acetylation activates transcription independent apoptotic activity of Abl
Source: Mol Cancer. 2011 Jul 22;10:88. doi: 10.1186/1476-4598-10-88 (PMC3157453; doi:10.1186/1476-4598-10-88)

## Slide 1
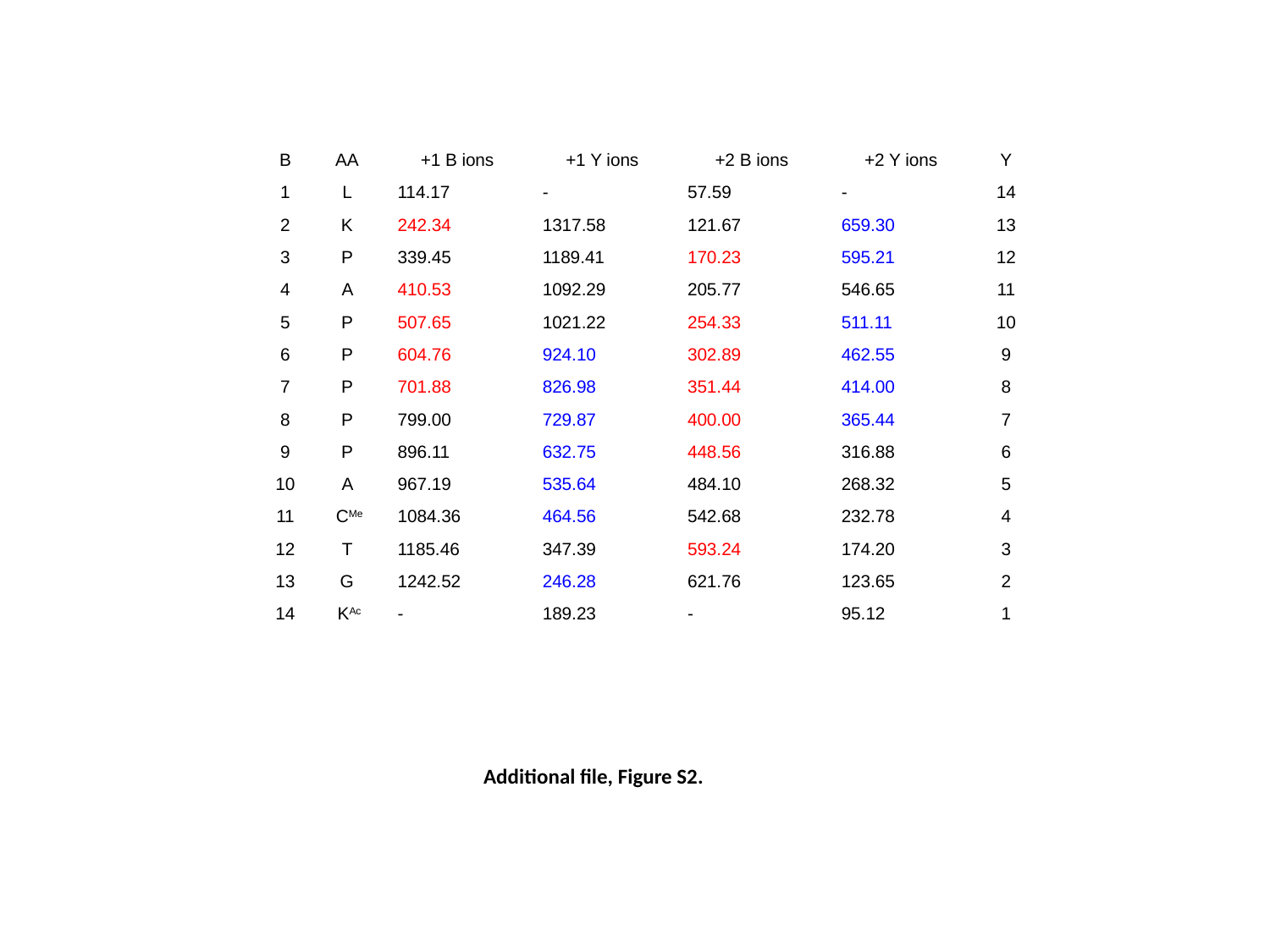

| B | AA | +1 B ions | +1 Y ions | +2 B ions | +2 Y ions | Y |
| --- | --- | --- | --- | --- | --- | --- |
| 1 | L | 114.17 | - | 57.59 | - | 14 |
| 2 | K | 242.34 | 1317.58 | 121.67 | 659.30 | 13 |
| 3 | P | 339.45 | 1189.41 | 170.23 | 595.21 | 12 |
| 4 | A | 410.53 | 1092.29 | 205.77 | 546.65 | 11 |
| 5 | P | 507.65 | 1021.22 | 254.33 | 511.11 | 10 |
| 6 | P | 604.76 | 924.10 | 302.89 | 462.55 | 9 |
| 7 | P | 701.88 | 826.98 | 351.44 | 414.00 | 8 |
| 8 | P | 799.00 | 729.87 | 400.00 | 365.44 | 7 |
| 9 | P | 896.11 | 632.75 | 448.56 | 316.88 | 6 |
| 10 | A | 967.19 | 535.64 | 484.10 | 268.32 | 5 |
| 11 | CMe | 1084.36 | 464.56 | 542.68 | 232.78 | 4 |
| 12 | T | 1185.46 | 347.39 | 593.24 | 174.20 | 3 |
| 13 | G | 1242.52 | 246.28 | 621.76 | 123.65 | 2 |
| 14 | KAc | - | 189.23 | - | 95.12 | 1 |
Additional file, Figure S2.

Supplement: Additional file 2 — MS/MS of the peptide ion at m/z 477.8, corresponding to the (M+3H)3+ species is shown. [file 1476-4598-10-88-S2.PPTX]

## Slide 1
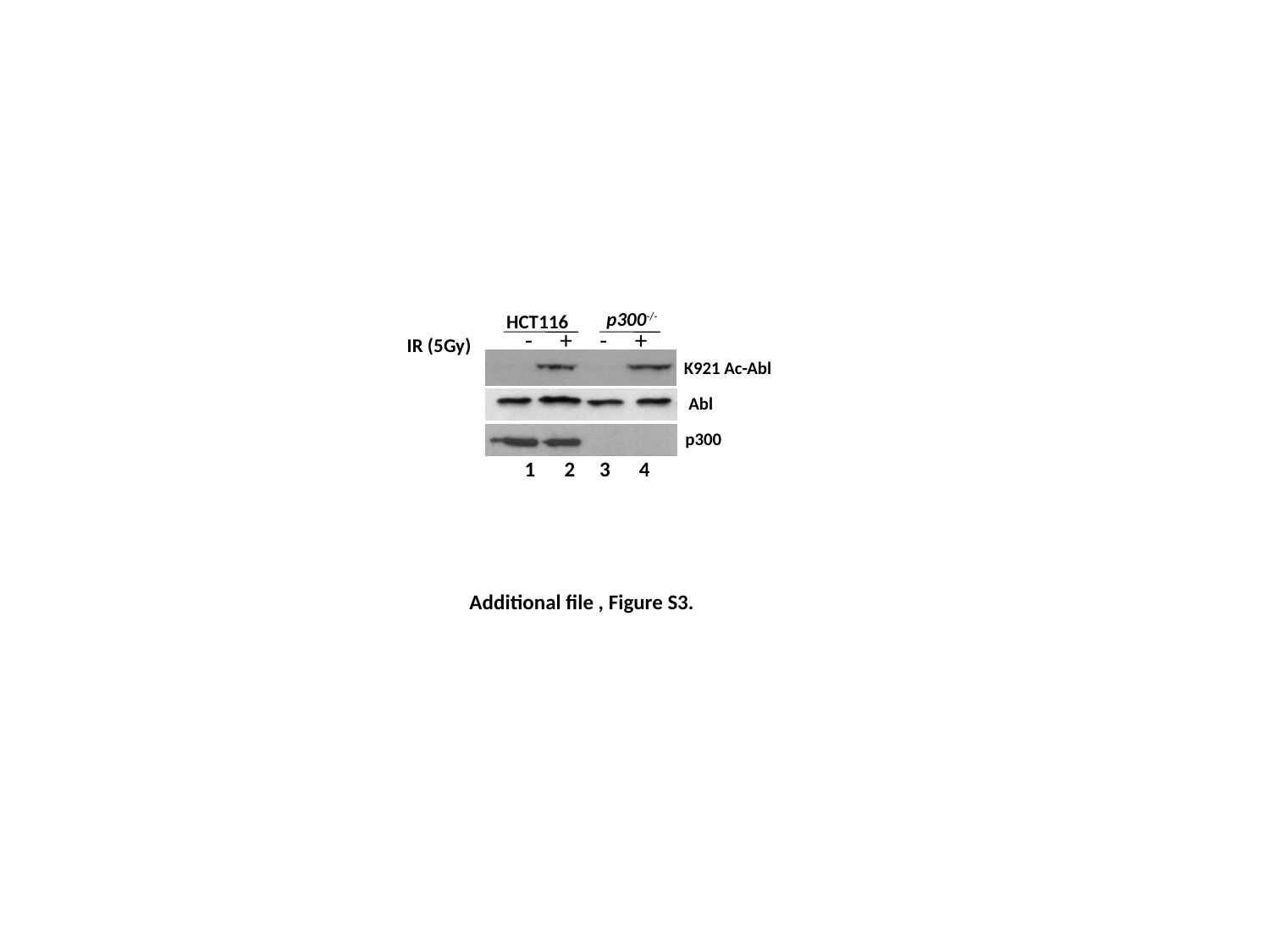

p300-/-
HCT116
- + - +
IR (5Gy)
K921 Ac-Abl
Abl
p300
1 2 3 4
Additional file , Figure S3.

Supplement: Additional file 3 — p300 acetyl-transferase is dispensable for IR-induced K921 Abl acetylation. Mock-treated and IR (5 Gy) exposed HCT116 and HCT116/p300-/-cells were harvested 4 h later. Lysates were adjusted for equal protein content and probed with K921 site specific anti-acetyl-Abl (top panel), anti-Abl (middle panel) or anti-p300 antibody (bottom panel). [file 1476-4598-10-88-S3.PPTX]
